# Aneurysmal Subarachnoid Haemorrhage in Pregnancy: A Case Series

**Published:** 2012-01-18

**Authors:** Maurizio Guida, Roberto Altieri, Valeria Palatucci, Federica Visconti, Renato Pascale, Marialuisa Marra, Giampiero Locatelli, Renato Saponiero, Rosalba Tufano, Francesca Bifulco, Ornella Piazza

**Affiliations:** 1 Department of Obstetrics & Gynecology, University of Salerno, Italy; 2 Department of Neurosurgery, University of Salerno, Italy; 3 Department of Operative Neuroradiology, University of Salerno, Italy; 4 Department of Anesthesiology and Intensive care unit, Federico II University of Naples; 5 Department of Anesthesiology and Intensive care unit, University of Salerno

**Keywords:** aneurismal subarachnoid hemorrhage, pregnancy, headache

## Abstract

Pregnancy is a recognized risk factor for aneurysmal subarachnoid hemorrhage (SAH). Headache is very frequent in normal pregnancy and it is a common sign shared between several intracranial diseases. We present a case series of 10 women in the third trimester of pregnancy admitted to our intensive care unit (ICU) with neurological signs and symptoms. 4 of these patients were diagnosed with SAH. Data in this study suggest that a timely diagnosis and an appropriate treatment is crucial for mother and baby.

Several pathological disorders affect the nervous system during pregnancy and puerperium. Someone are specific disorders of pregnancy, others are common neurologic disorders that are most frequent in this period. Headache is very common during pregnancy. Nevertheless, headache remains the commonest symptom of intracranial disease and the development of acute headache should be taken seriously [1]. Several conditions during pregnancy present with migraine, seizures and focal neurological deficits. Differential diagnosis may result very difficult and should be performed on history, basic labs and neuroimaging studies. Delay of diagnosis may result in poor outcome. There is an association between pregnancy and risk of stroke. The incidence of SAH is increased during pregnancy and relatively few studies have been conducted to document the risk [2, 3]. SAH is the only type of stroke with female predominance, suggesting that reproductive factors may play a role in the etiology [4]. This pathology occurs more often in primiparae and in the third trimester of pregnancy. Okamoto and al found an increased risk of SAH with an earlier age of menarche and with nulligravity [5]. The hemodynamic, coagulation and endocrine changes play a central role in the growth and rupture of aneurysms, changing arterial and venous intima and media organization [6,7,8]. During pregnancy and puerperium, rupture of an intracranial aneurysm still remains the commonest cause of SAH [1]. The reported prevalence of SAH during pregnancy is 1/10.000 patients. This translates to a prevalence five times higher than in non pregnant women [9]. With significant improvements in prenatal care and management of deliveries, non obstetric causes of maternal death such as aneurysmal subarachnoid haemorrhage are likely to become increasingly significant [10].There are no differences in the clinical course of SAH among pregnant and non-pregnant patients.

## Methods

We report a case series of 10 pregnant women with neurological disorders admitted at ICU of our hospital between September 2008 and September 2011. All the patients were studied with CT scan or MRI. All the patients were in the third gestational trimester with previous normal pregnancy. Presence of aneurysm in patients with SAH was confirmed with Digital Subtraction Angiography (DSA). Glasgow coma scale (GCS) was performed to assess neurological status; the Hunt and Hess grading system was carried out in patients with SAH [11]. All the patients who underwent treatment for ruptured aneurysms were classified based on the Fischer's grading system into grades 1-4 (Group 1→ No blood detected; Group 2 → Diffuse deposition of subarachnoid blood, no clots and no layers of blood greater than 1 mm; Group 3 → Localized clots and/or vertical layers of blood 1 mm or greater in thickness; Group 4 → Diffuse or no subarachnoid blood, but intracerebral or intraventricular clots are present) [12].

## Results

Table 1 summarizes data of our patients. All patients presented severe headache. Six out of our patients presented seizures; three out of these presented blurred vision; two out of these presented focal neurological signs; one out of these presented vomit without nausea; five out of these patients presented consciousness alterations. All the patients had a GCS > 8 at admission. We performed emergency cesarean section in all patients and all babies are survivors. 8 patients were studied with CT scan, 6 patients were studied with MRI. In 5 cases neuroimaging studies demonstrated Posterior Reversible Encephalopathy Syndrome (PRES), in 1 case ischemic stroke, in 4 cases SAH. Table 2 shows data of patients with SAH. We analyzed patients with SAH.

### Case 1

**Figura 2:**
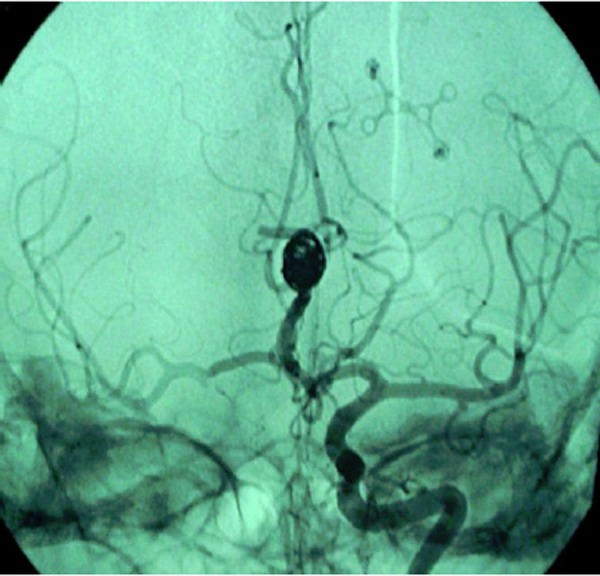
the same aneurysm after embolization

The female patient aged 37, 34-week pregnant, arrived in emergency room of our hospital reporting a sudden and severe headache accompanied by vomiting. Upon admission to the hospital, the patient did not present neurological signs, except a mild neck rigidity. The woman presented Hunt-Hess grade II. CT scan showed that the hemorrhage was located on the cisterns of the base, extending to the sylvian and interemispheric fissures. The patient was diagnosed with SAH. After delivery induction the woman underwent Digital Subtraction Angiography (DSA). DSA showed a large (20 mm) saccular aneurysm of the post-communicating segment of anterior cerebral artery (A2) (fig. 2). She was not undergone to embolization for diffuse vasospasm of entire ipsilateral carotid artery network. The indication of urgent surgery was given for presence of hematoma. A favorable postoperative course and a recovery of neurological and general conditions followed, without residual disability, as assessed by the Functional Independence Measure (FIM) scale. Angiographic control at one year away from the event showed the recanalization of aneurysm. The patient was therefore subjected to endovascular treatment with Guglielmi Detachable Coil (GDC); 10 F microcatheter; microguide 10 F. The patient recovered well. After one year, DSA showed no aneurysm recanalization (fig. 3).

### Case 2

The female patient 34-year-old, 37-weeks pregnant, arrived in emergency room of our hospital with confusion and moderate right hemiparesis. Upon admission to the hospital, the woman presented Hunt-Hess grade III. CT scan showed a diffuse subarachnoid haemorrhage and localized clots.The patient was diagnosed with SAH. After caesarean section, the woman underwent DSA that shows a small (10 mm) saccular aneurysm of the middle cerebral artery (MCA) bifurcation. She underwent embolization. Patient died two days after.

### Case 3

The female patient 41-year-old, 32-weeks pregnant, arrived in emergency room of our hospital with lethargy and mild focal neurological deficit. Woman presented Hunt-Hess grade III. CT scan showed subarachnoid hemorrhage and vertical layers of blood. The patient was diagnosed with SAH. After caesarean section, the woman underwent DSA that showed a large (15 mm) saccular aneurysm of the anterior communicating artery (ACA). She underwent embolization and died the day after.

### Case 4

The female patient 28-year-old, 33-weeks pregnant, arrived in emergency room of our hospital with seizures. After stabilization, patient referred severe headache and presented mild nuchal rigidity. Woman presented Hunt-Hess grade II. CT scan showed diffused blood layers less than 1 mm thick. The patient was diagnosed with SAH. After delivery induction the woman underwent DSA that showed a large (20 mm) saccular aneurysm of basilar artery (BA) apex. She underwent embolization. Patient recovered well general condition but remained with mild neurological impairment.

## Discussion

SAH is a very rare complication in pregnancy. SAH during pregnancy may be confused with eclampsia, because of high blood pressure, seizures and consciousness alterations. The confirmation of the diagnosis is made with neuroimaging (CT scan, MRI and cerebral angiography). CT scanning exposes the foetus to radiation but the benefits of CT scanning greatly outweigh the risks in this situation. Therefore, the evaluation of pregnant patient arriving with headache demands a full general and neurological examination together with an assessment of mental status (GCS). It may be necessary to include examination of the teeth, eyes, paranasal sinuses and urine and various investigative procedures may be indicated, depending on the initial clinical impression. If intracranial disease is suspected on the bases of history or presence of neurologic signs, the need for CT must be decided on an individual bases. The use of Magnetic Resonance imaging in pregnancy is controversial. Some animal studies demonstrated that MRI exposure may affect fetal development. There are not these evidences in human studies. When CT and MR images are negative and clinical findings are very suggestive of SAH lumbar puncture should be performed.

SAH from aneurysmal rupture should be managed from neurosurgeons, but in pregnant women management is multidisciplinary (intensivist, neurosurgeon, neuroradiologist, obstetric, neonatologist). It is very important to obtain a prompt neurosurgical consultation.

In our experience for the pregnancy management it is better to perform emergency cesarean section before SAH treatment except in early pregnancy when patient should be treated like not pregnant [13]. In these cases when aneurysm is clipped the pregnancy can be allowed to progress to term and the vaginal delivery is preferred by most clinicians [13,14]. Caesarean section would be indicated in several circumstances: when the clinical state of mother is severe (coma, brainstem damage), if the aneurysm is diagnosed at term of labour, if the interval between the neurosurgical treatment of aneurysm and labour is less than 8 days [15].

In aneurysmal SAH clipping of the aneurysm is recommended. In our cases series we performed intravascular embolization in all cases but one. We treated one case with surgery because severe vasospasm. Several studies in the last decade have established the efficacy and safety of endovascular coiling in the treatment of cerebral aneurysms [16]. Successful endovascular coil treatment of aneurysms during pregnancy has been reported [17,18].

## Conclusions

While rare, aneurysmal SAH during pregnancy can be devastating for both mother and child, only an early diagnosis and appropriate treatment can give at mother and son a possibility to survive. It is therefore imperative that all obstetricians and gynecologists know this nosocomial entity so that a high index of suspicion is maintained when pregnant women presents with acute headache particularly if it is accompanied by neurological signs like nuchal rigidity, palsy or seizures. It is just necessary a multidisciplinary team to manage this emergency. The involvement of an experienced neurosurgeon and a neuroradiologist plays a central role in managment of SAH in pregnant women. Endovascular coiling treatment of cerebral aneurysms is a minimally invasive and efficient method to reduce the rate of rebleeding.

## Figures and Tables

**Figura 1:**
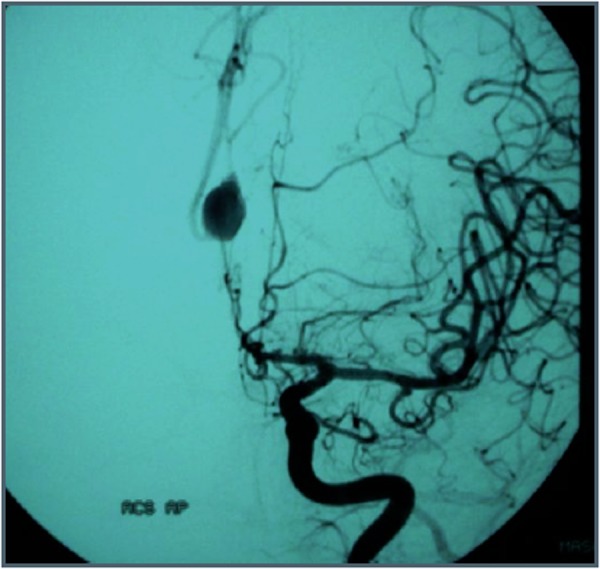
saccular aneurysm of A2

**Table 1:** Data of patients (GA: gestational age)

Patient	Age	GA	Symptoms	Diagnosis	Neuro-imaging
1	37	34	Headache, vomiting, neck rigidity	SAH	CT, DSA
2	36	36	Seizures	PRES	MRI
3	38	35	Seizures, blurred vision	PRES	MRI
4	34	37	Confusion, moderate hemiparesis	SAH	CT, DSA
5	39	34	Seizures, confusion	PRES	CT, MRI
6	41	32	Lethargy, focal signs	SAH	CT, DSA
7	41	38	Seizures, headache	PRES	CT, MRI
8	28	33	Seizures, headache, nuchal rigidity	SAH	CT, DSA
9	30	35	Confusion, seizures, blurred vision	PRES	CT, MRI
10	27	27	Blurred vision, confusion	Ischemic stroke	CT, MRI

**Table 2:** This table summarizes data of our patient.

Patient	Hunt-Hess grade	Aneurism location	Aneurism size (mm)	Treatment	Mother outcome
1	II	A2	20	surgery	Complete recovery
2	III	MCA bifurcation	10	GDC	Died
3	III	ACA	15	GDC	Died
4	II	BA apex	20	GDC	mild neurological impairment
